# *Fusarium* Mycotoxins in Maize Field Soils: Method Validation and Implications for Sampling Strategy

**DOI:** 10.3390/toxins14020130

**Published:** 2022-02-09

**Authors:** Kilian G. J. Kenngott, Julius Albert, Friederike Meyer-Wolfarth, Gabriele E. Schaumann, Katherine Muñoz

**Affiliations:** 1 Group of Environmental and Soil Chemistry, Institute for Environmental Sciences (iES) Landau, University of Koblenz-Landau, Fortstraße 7, 76829 Landau, Germany; kenngott@uni-landau.de (K.G.J.K.); albert.j@uni-landau.de (J.A.); schaumann@uni-landau.de (G.E.S.); 2 Julius Kühn Institute (JKI), Federal Research Centre for Cultivated Plants, Institute for Plant Protection in Field Crops and Grassland, Messeweg 11/12, 38104 Braunschweig, Germany; friederike.meyer-wolfarth@julius-kuehn.de; 3 Group of Organic and Ecological Chemistry, Institute for Environmental Sciences (iES) Landau, University of Koblenz-Landau, Fortstraße 7, 76829 Landau, Germany

**Keywords:** mycotoxins, *Fusarium*, soil, deoxynivalenol, nivalenol, environment, monitoring strategies, validated method, maize field

## Abstract

While mycotoxins are generally regarded as food contamination issues, there is growing interest in mycotoxins as environmental pollutants. The main sources of trichothecene and zearalenone mycotoxins in the environment are mainly attributed to *Fusarium* infested fields, where mycotoxins can wash off in infested plants or harvest residues. Subsequently, mycotoxins inevitably enter the soil. In this context, investigations into the effects, fate, and transport are still needed. However, there is a lack of analytical methods used to determine *Fusarium* toxins in soil matrices. We aimed to validate an analytical method capable of determining the toxins nivalenol (NIV), deoxynivalenol (DON), 15-acetyl-deoxynivalenol (15-AcDON), and zearalenone (ZEN), at environmentally relevant concentrations, in five contrasting agricultural soils. Soils were spiked at three levels (3, 9 and 15 ng g^−1^), extracted by solid-liquid extraction assisted with ultrasonication, using a generic solvent composition of acetonitrile:water 84:16 (v:v) and measured by LC–HRMS. Method validation was successful for NIV, DON, and 15-AcDON with mean recoveries > 93% and RSD_r_ < 10%. ZEN failed the validation criteria. The validated method was applied to eight conventionally managed maize field soils during harvest season, to provide a first insight into DON, NIV, and 15-AcDON levels. Mycotoxins were present in two out of eight sampled maize fields. Soil mycotoxin concentrations ranged from 0.53 to 19.4 ng g^−1^ and 0.8 to 2.2 ng g^−1^ for DON and NIV, respectively. Additionally, we found indication that “hot-spot” concentrations were restricted to small scales (<5 cm) with implications for field scale soil monitoring strategies.

## 1. Introduction

*Fusarium* is a genus of filamentous fungi, of which some species are known to be pathogenic to various plants, including cereal crops. These pathogenic species cause several serious plant diseases, such as stalk rot, which is globally the main reason for crop loss in maize production [1]. Furthermore, they are able to produce toxic secondary metabolites of the mycotoxin families, Type B trichothecene and zearalenone [2]. Among these mycotoxin groups, deoxynivalenol (DON), nivalenol (NIV), and zearalenone (ZEN) are of particular interest due to the frequent occurrence and the concentration levels in harvest samples and freshwater streams [3,4,5,6]. Type B trichothecenes, such as DON and NIV, are toxic to humans and animals [7]. ZEN is an endocrine disrupting substance [8], which may have estrogenic effects on fish when transported into rivers [9]. For these reasons, concentrations of mycotoxins in food and feed are regulated in many countries for the safe commercialization of food and feed commodities [10,11], which is why there are many analytical methods for various food matrices [12,13].

Mycotoxins are usually regarded as food contamination problems but there is growing interest in mycotoxins as soil and aquatic pollutants of emerging concern. Some studies have detected mycotoxins in soil, surface water, and sewage sludge [4,14,15,16,17,18,19]. Diffusive sources of DON, NIV, and ZEN in the environment are mainly attributed to *Fusarium* infested fields, where mycotoxins can be washed off the infested plants entering soil and drainage water [4,20]. Another source can be harvesting residues leaching out mycotoxins on the fields [21,22]. In both cases, mycotoxins will initially enter the soil, where the effects, fate, and transport are scarcely investigated.

To assess mycotoxin occurrence and its fate in soil, suitable quantification methods are imperative. However, the available methods from food analyses cannot be easily applied to soil due to the complexity and heterogeneity of the soil matrices and the much lower expected concentration levels in soil (a lower $ng\;g^{-1}$ scale) compared to a food and feed ($\mu g\;g^{-1}$ scale, [6]). The most commonly applied analytical method for mycotoxin analysis is liquid chromatography with mass spectrometry (LC–MS) [13,23,24]. However, LC–MS measurement of mycotoxins is sensitive to matrix effects [25,26], which is caused by ions and organic acids contained in a sample, leading to signal enhancement or suppression. The potential load of such a matrix in soil samples is mainly described by physicochemical parameters, such as soil pH, soil organic carbon, and clay content. These parameters can be highly variable even at small field scales [27,28], leading to the necessity of the matrix effect assessment of individual samples. Therefore, extraction and exact quantification of mycotoxins in soils is challenging [29]. The usefulness of isotopically-labeled internal standards has been reported on in several studies [20,30,31]. However, it remains unclear whether this tool is suitable to compensate matrix effects of different soils, leading to a reduced number of matrix-matched calibrations.

Some methods were already reported to be effective for extraction and quantification of trichothecenes from sandy loam, loamy silty sand, and sandy loamy silt of a strawberry field [17,18]. Mortensen et al. [32] established an extraction method for ZEN using three different agricultural soils with varying texture and carbon content, but used spiking concentrations (30–60 ng $g^{-1}$) that were clearly higher compared to natural levels (0.6–1.1 ng $g^{-1}$, [19]; 0–7.5 ng $g^{-1}$, [16]). In this study, we validate an extraction and quantification method with the aim of being suitable for selected Type B trichothecenes (NIV, DON, 15-AcDON) and ZEN in various agricultural soils at environmentally realistic concentrations.

Another challenge in soil mycotoxin analysis is the sampling strategy. Natural infestations of plants may not be homogeneously distributed over the field [33]. Randomly taken soil cores that are pooled together may lead to dilution and consequently no detection of mycotoxins or underestimation of mycotoxin concentrations. Furthermore, small scale concentration patterns may also hamper mycotoxin detection. Mycotoxins that are produced above ground in the plant can be washed off by the rain and rinse down the maize plant stem to the soil surface. Furthermore, *Fusarium* is able to grow on harvest residues left on top of the soil. Sub-soil mycotoxin production is yet not investigated, but it seems to be possible [19]. Both cases would lead to a “hot-spot” concentration close to individual infested plants or harvest residues, i.e., maize stubble. Therefore, the sampling strategy on the filed scale is crucial for reliable mycotoxin detection. Knowledge about these hot-spot soil mycotoxin concentrations is necessary to assess the potential dose-dependent effect on the soil microbiome or meso organisms. Additionally, plant uptake of mycotoxins has been shown for ZEN [34], ochratoxin A [35], DON [36], and was shown to be dose-dependent for aflatoxins [37]. The species *F. verticillioides* and *F. graminearum* are frequently found on maize in Germany [38]. These species are able to produce various type A and type B trichothecenes, of which DON and NIV were found most frequently (>60%) in the maize sampled in Germany [38,39]. Additionally, these mycotoxins were also frequently found in freshwater streams of basins with widespread maize production [4]. Therefore, these mycotoxins are of particular interest for environmental soil sampling.

We hypothesize that *Fusarium* toxins predominately enter the soil via washing off plants, leading to elevated soil concentrations (hot-spots) in the soil. These hot-spots can be identified by screening the pooled samples for increased levels. We applied the validated method on conventionally managed maize field soils that were not artificially infested with *Fusarium*, to provide the first insight into natural mycotoxin levels and to derive sampling strategies for future research.

## 2. Materials and Methods

### 2.1. Chemicals

HPLC grade acetonitrile used for extraction was supplied by Carl Roth (Karlsruhe, Germany). LC–MS grade methanol and formic acid for mobile phase and standard preparations were supplied by Fisher Scientific (Schwerte, Germany). Ultrapure water was used throughout the whole study (Thermolyne EASYpure II, EnviroFALK PharmaWaterSystems, Leverkusen, Germany). Non-labeled standards of NIV, DON, 15-AcDON, ZEN, and isotopically labeled standards of ^13^C_15_-DON and ^13^C_15_-NIV, all dissolved in acetonitrile, were supplied by Biopure^TM^, Romer Labs (Butzbach, Germany).

Non-labeled standards were combined to one standard mix stock solution containing 1000 ng m$L^{-1}$, which was further diluted with methanol to spiking stocks containing 150, 450 and 750 ng m$L^{-1}$ of each compound, respectively. All calibrations were prepared from the spiking stocks, prior to the measurement. Isotopically-labeled standards were mixed and diluted with methanol to a spiking stock containing 250 ng m$L^{-1}$ of each compound, respectively. The same isotopically labeled standard stock was used throughout the whole study and was spiked to each individual sample and calibration prior to measurement.

### 2.2. Soil Samples for Method Validation

Five soils were selected representing a broad spectrum of agricultural soils, to ensure robustness of the analytical procedure. Clay and soil organic carbon content are assumed to be the most influential parameters for method performance. Due to the great surface and number of sorption sites, these soil fractions bear the greatest potential in retaining the mycotoxins [40]. Furthermore, these soil fractions contain the majority of extractable organic and inorganic molecules, which may cause matrix effects during the measurement. The soils used for method validation were the reference soils LUFA2.4, LUFA6S supplied from LUFA Speyer (Speyer, Germany), RefeSol01A and RefeSol02A supplied by Fraunhofer IME (Schmallenberg, Germany), and one natural maize field soil, supplied by JKI (Braunschweig, Germany). The reference soils were air-dried and sieved (2 mm). Selected soils can be classified as sandy loam (LUFA2.4), clayey loam (LUFA6S), silty loam (RefeSol01A), and clayey silt (RefeSol02A), see Table 1 for detailed physicochemical parameters. The non-reference JKI soil was sampled in September 2019 from four non-infested sub-plots within one maize field located in Braunschweig (Germany), where 10 samples of approximately 40 mL of soil volume were taken from the topsoil (12 cm) of each sub-plot using an auger. Samples were frozen and sent to Landau (Germany), where they were pooled, freeze-dried, sieved using a 2 mm mesh size stainless steel sieve, and homogenized.

### 2.3. Environmental Monitoring Strategy

To validate the applicability of the proposed method, mycotoxins were investigated in eight conventionally managed maize field soils located around Geinsheim, Germany. Sampling was done in September/October 2020 during harvest season when plants were dried for harvest (BBCH-scale 97). Since fields were used for forage maize production, none were irrigated for several weeks before sampling. Sampling of conventionally managed maize fields was designed to detect potential concentration gradients and to suggest sampling strategies for future monitoring studies. Since many fields potentially do not contain any mycotoxins, analysis of all individual samples may lead to a large number of negative results. To reduce the number of samples, samples pooled by plot and position were analyzed, resulting in eight composite samples per field. When elevated levels of mycotoxins were detected in the pooled samples, individual samples of the respective plot and position were analyzed to assess small-scale concentration patterns (10 samples per plot and position, 3 replicates per sample).

**Figure 1:**
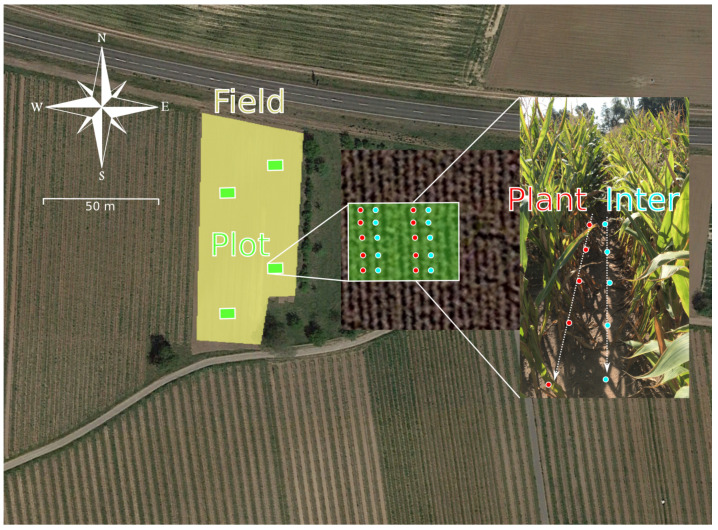
Sampling scheme for naturally infected maize field soils. Four plots were selected on each of the eight fields. Each plot was sampled on two positions, “plant” and “inter” row, represented by red and blue dots, respectively. Each position was sampled five times in two rows of each Plot. Map source: *©* Google Earth Pro.

A sampling scheme is presented in Figure 1. Field sizes ranged between 2700 and 30,300 m${}^{2}$(median 7700 $m^{2}$). On each field, four sub-plots (30 $m^{2}$) were defined at equally distributed positions within the field. To detect potential concentration differences depending on the sampling position distance to the plant, we sampled on two positions, i.e., between plants (“plant”) and inter-row (“inter”). The mean distances between the plants and plant rows were 20 ± 10 cm and 76 ± 7 cm, respectively. Each position was sampled five times (1 m distance) in two rows (approximately 6 m distance) on each sub-plot, resulting in ten samples per position and sub-plot. Sampling was done by horizontally pushing a cylindrical plastic container (5 cm diameter) into the soil, thereby scratch-sampling a topsoil volume of about 80 mL. The first scratch sample of each sampling point was collected in a plastic bag, resulting in a pooled sample for each position and sub-plot. The second scratch sample was kept in the respective plastic cup, closed and stored at −20 ${}^{\circ }C$ in the lab until further preparation.

### 2.4. Soil Characterization

The soils were characterized by soil texture, total soil carbon, soil pH, and water content when field fresh samples were available. Reference soils were characterized by the supplier. Soil pH and water content of non-reference soils were measured in plot-wise pooled samples. Soil texture was determined according to DIN 19682-2. Total soil carbon content was measured by dry combustion in tin foils using an elemental analyzer (vario MICRO cube, Elementar Analysensysteme GmbH, Langenselbold, Germany). Water content was measured gravimetrically as mass loss after 48 h of freeze-drying. Soil pH was measured electrochemically in a 0.01 mol $L^{-1}$ CaCl_2_ solution (1:5 ratio soil:solution). Physicochemical properties of the soils are shown in Table 1.

### 2.5. Soil Spiking and Extraction

Fractions of 5 g air-dried reference soils were weighed in 50 mL centrifuge tubes and spiked with 100 μL spiking standards or pure methanol to obtain three soil concentration levels at 3, 9 and 15 ng $g^{-1}$ and blank control. Ten replicates were prepared for each reference soil, concentration level, and blank control. Additionally, the method was tested with the JKI maize field soil samples, which were spiked similarly at the levels 3 and 9 ng $g^{-1}$. Extraction of the JKI soil samples was repeated three times on different days to assess within-field and inter-day variability. Spiking levels were chosen to be close to the expected limit of detection and in the same order of magnitude as natural soil concentrations (i.e., 2–28 ng $g^{-1}$ reported in [17,18]). After spiking, all samples were vortexed for 10 s and left open under the fume hood for 30 min to ensure homogeneous distribution of the sample and methanol evaporation.

Samples were extracted using 15 mL acetonitrile:water (84:16 v:v) as it is a commonly used solvent mixture for multi mycotoxin extraction in different food and feed matrices [13]. The soils were mixed with the extraction solvent for 30 min at 180 rpm in an orbital shaker. After 15 min in an ultrasonic bath, samples were centrifuged for 15 min at 3500 rpm. Aliquots of 4 mL of each extract were evaporated to dryness at 40 ${}^{\circ }C$ under a gentle nitrogen stream. The samples were reconstituted with 0.8 mL 1:9 methanol:water mixture by vortexing for 10 s and ultrasonication for 10 min. Concentrated extracts were filtered through 0.2 μm PET syringe filters. Aliquots of 90 μL of the filtered extracts were mixed with 10 μL isotopically labeled standard mixture before measurement.

### 2.6. LC–HRMS Analysis

Separation and quantification of mycotoxins was carried out by a liquid chromatography high resolution mass spectrometry (LC–HRMS) Thermo Scientific^®^ system equipped with an Accela Quaternary^®^ pump, an Exactive^®^ Orbitrap MS detector (Thermo Fisher Scientific, Waltham, MA, USA), and a Hypersil GOLD™ C18 column with dimensions 100 × 2.1 mm, and 1.9 μm particle size (Thermo Fisher Scientific, Waltham, USA). The mobile phase consisted of methanol (Solvent A) and water (Solvent B) both conditioned with 0.1% formic acid, in the following gradient program: 0–2 min 19% Solvent A; 2–6 min 19–100% Solvent A; 6–11 min 100% Solvent A; 11–12 min 100–19% Solvent A; 12–15 min 19% Solvent A. Flow rate was constant at 0.2 mL $\min^{-1}$. Injection volume was 10 μL. Electron spray ionization was performed in positive and negative mode simultaneously at a capillary temperature of 275 ${}^{\circ }C$. Electronic settings were as follows: spray voltage 4 kV, capillary voltage 25 V, tube lens voltage 75 V, and skimmer voltage 14 V.

The target masses of all mycotoxins with the respective adduct and retention time used for HRMS detection are shown in Table 2. NIV, DON, and the respective isotopically labeled standards were measured in negative modes, as the formic acid adduct. ZEN was measured in a negative mode, as the [M-H]${}^{-}$ adduct. Parameters for NIV, DON, and ZEN matched well with data reported in the Thermo Scientific application note 51,961 by [41], using a very similar technical setup.

The masses 361.1257 *m/z* and 356.1705 *m/z* measured in positive mode, were found suitable for 15-AcDON detection and were attributed to the respective sodium and ammonium adduct. The sodium adduct has been described in literature several times [42,43,44], and was used as a qualifier, while the ammonium adduct was used as a quantifier due to the slightly higher signal intensities.

Matrix matched calibrations for method validation were measured at concentration levels 0.8, 2, 4, 8, 20 and 80 ng m$L^{-1}$. Field soil samples were quantified in a narrower range to adapt to environmentally realistic concentrations (0.75, 1.5, 3.75, 7.5, 15, 22.5 and 30 ng m$L^{-1}$) in methanol:water (20:80, v:v). Matrix matched calibration and internal standard correction were applied for recovery experiment quantification. Internal standard correction was applied for environmental sample quantification.

### 2.7. Method Validation Criteria

In line with the Eurachem guide [45], method validation criteria were selectivity, linearity of the working range, matrix effect, trueness (bias), precision, method limit of detection (LOD), and quantification (LOQ). Selectivity was ensured by identifying characteristic *m/z* ratios, comparison of *m/z* ratios with literature and comparison of chromatograms with internal and external standard measurements. Linearity of the working range was assessed by visual inspection of the calibration curve and calculation of the adjusted coefficient of determination R^2^_adj_ after linear regression. Assumption of normality and homoscedasticity were evaluated with QQ and residual vs. fitted plots [46]. A weighted least squares linear regression (WLS) was performed according to Almeida et al. [47] when assumption of homoscedasticity was not met. Briefly, the increase in variation, i.e., heteroscedasticity, may often be described as a function of concentration. Therefore, by selecting an appropriate weighing factor, the WLS aims to account for greater influence of greater concentrations on the regression and minimize the sum of relative errors instead of the sum of squares. As suggested by the authors, the following weighing factors were tested: 1/x^0.5^, 1/x, 1/x^2^, 1/y^0.5^, 1/y and 1/y^2^ with x as the nominal concentration and y as the peak area. The matrix effect was assessed as the signal suppression enhancement ratio (SSE) and calculated as the deviation of matrix-matched calibration slopes from the solvent calibration slope, expressed in percent [29]. Additionally, the signals of NIV and DON were related to the respective internal standard signal. These standardized signals were used for SSE recalculation to evaluate the use of internal standards to eliminate the matrix effect, i.e., minimizing SSE. Trueness (in terms of mean spike recovery) and precision (in terms of relative standard deviation of recoveries, RSD_r_) were calculated as described in Magnusson and Örnemark [45]. According to the Commission of the European Communities [48], the acceptable recovery range for DON at concentrations in the range 100–500 ng $g^{-1}$ in foodstuffs is 60–110% with RSD_r_ < 20%. Since method performance criteria for lower concentrations or other similar mycotoxins, such as NIV and 15-AcDON, are not defined, we use the same as for DON. The criteria for ZEN at concentrations below 50 ng $g^{-1}$ are defined as 60–120% recovery and RSD_r_ < 20%

The method LOD and LOQ were calculated based on the lowest recovery spiking level of 3 ng $g^{-1}$ (the near instrumental limit of quantification). Standard error of the 10 replicates was multiplied by 3 and 10 to calculate LOD and LOQ, respectively [45].

### 2.8. Data Evaluation

All data evaluations and presentations were performed using the software R [49], including the package ‘data.table’ and ‘ggplot2’ [50,51]. Assumption of normality and homoscedasticity of linear model residuals were evaluated with QQ and residual vs. fitted plots [46]. When linear models for calibration showed heteroscedasticity of residuals, the best weighting factor for WLS calibration was selected using the function *weight_select* and WLS was performed using the function *calibration* from the package ‘envalysis’ [52]. To reduce the matrix effects, peak areas of NIV and DON were divided by the peak area of the respective isotopically labeled internal standard. To assess within-field and inter-day variability in the JKI soil extraction, the effects of predictor variables “extraction batch” and “sub-plot” on the recovery of individual mycotoxins were tested using a two-way analysis of variance models (F-ANOVA). The effect of the predictor variable “spiking level” on mean recoveries of respective mycotoxins was tested via the linear mixed effect models (LME) using the function *lmer* from the package ‘lmerTest’ [53]. To account for the variability caused by different reference soils, the factor “soils” was included in the models as a random effect. The effect of predictor variables “clay” and “soil organic carbon content” on mean recoveries was tested via LME, including “spiking level” as a random effect to account for potentially caused variation. Degrees of freedom and F-statistics were performed via the Kenward–Roger approximation, according to [54], using the package ‘lmerTest’ and ‘pbkrtest’ [53,54].

## 3. Results

### 3.1. Soil Characterization

Soil physicochemical properties are shown in Table 1. Soil types of field samples varied greatly from sand (Field 1) to clay (Field 8). The lowest soil pH was found in Fields 2 and 4, with values around 5.8. All other field soil pH values ranged from 6.6 to 7.5. Total carbon content in field samples ranged from 0.68 to 2.4.

### 3.2. Linearity and Matrix Effect

The R^2^_adj_ for all matrix-matched and solvent calibrations was well above 0.99, with one exception for 15-AcDON with 0.987. A total of 3 out of 16 calibrations for reference soils had heteroscedastic residues and WLS was applied.

**Figure A1:**
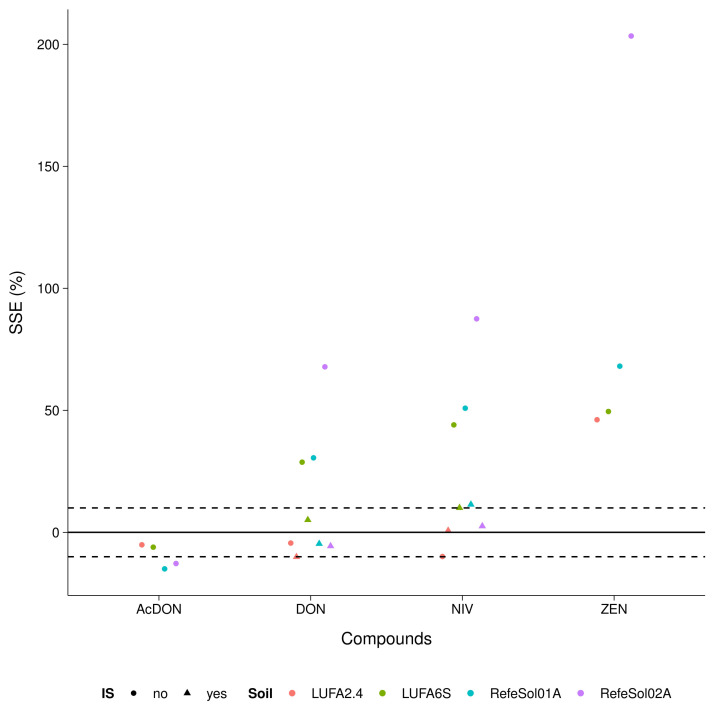
Comparison of the matrix-matched calibration slope with the calibration slope measured in pure solvent. The matrix effect is calculated as the difference between slopes divided by the solvent slope and expressed in percent (relative deviation). Different shapes (IS) indicate whether signals were standardized with an internal standard (triangle) or not (dot). Colors refer to the different matrices extracted from reference soils. Dashed lines denote the 10% interval around zero signal suppression/enhancement (straight line).

SSE was highly variable between soils and compounds (Figure A1). For all matrices, a minor SSE of −10±5% was observed for 15-AcDON. SSE of DON and NIV ranged from −4% to 68% and −10% to 88%, respectively. This was clearly reduced by the use of isotopically labeled internal standards to SSE of −4± 6% for DON and 6± 5% for NIV. Greatest variation in SSE between soils was observed for ZEN with 90± 80%.

No systematic effect of clay or soil organic carbon content on matrix effect was observed.

### 3.3. Trueness and Precision

**Figure 2:**
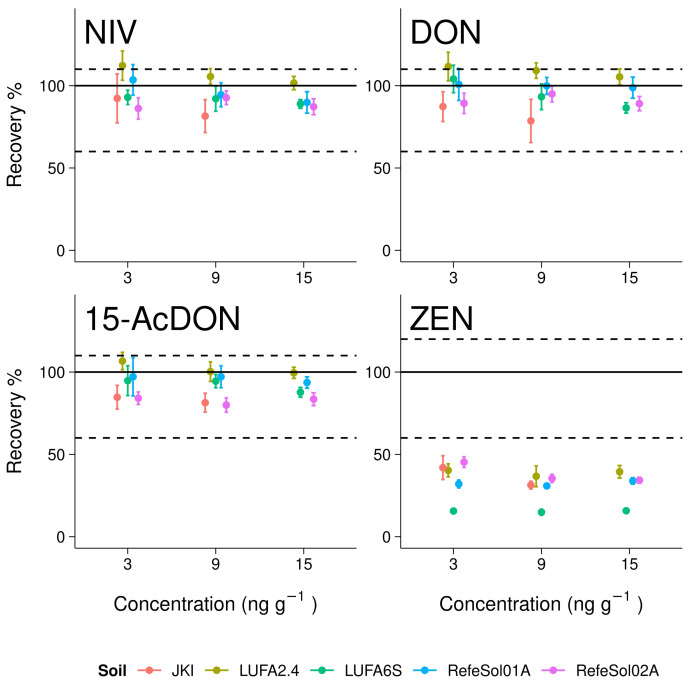
Recovery of nivalenol (NIV), deoxynivalenol (DON), 15-acetyl deoxynivalenol (15-AcDON), and zearalenol (ZEN) from different reference soils (LUFA Speyer and IME Fraunhofer) and one maize field soil (JKI). The dashed lines refer to the required performance criteria, according to Commission Regulation (EC) no. 401/2006.

Recovery of NIV, DON, and 15-AcDON was well above the required limit of 60% for all reference soils (Figure 2, Table A1). Mean recovery of NIV and DON was within the required range of 60–110% in 94% of all reference soil–concentration level combinations. There was only one exception for the lowest spiking level in LUFA2.4 soil, with mean recoveries of 112.2% and 111.6% for NIV and DON, respectively. Mean recoveries of 15-AcDON were always within the required range. Mean recoveries of ZEN ranged from 14 to 45% and, thus, never met the requirements. ZEN was therefore excluded from further evaluations.

There was a slight but significant trend towards lower recoveries at higher spiking concentrations for NIV (*p* = 0.035, F-test, df = 7), DON (*p* = 0.059, F-test, df = 7), and 15-AcDON (*p* = 0.029, F-test, df = 7). The variation of recovery significantly increased with decreasing spiking levels for NIV (*p* = 0.046, F-test, df = 7) and DON (*p* = 0.01, F-test, df = 7). The RSD_r_ was below the required limit of 20% for NIV, DON, and 15-AcDON in all reference soils and even below 10% for the majority with one exception for 15-AcDON.

**Figure A2:**
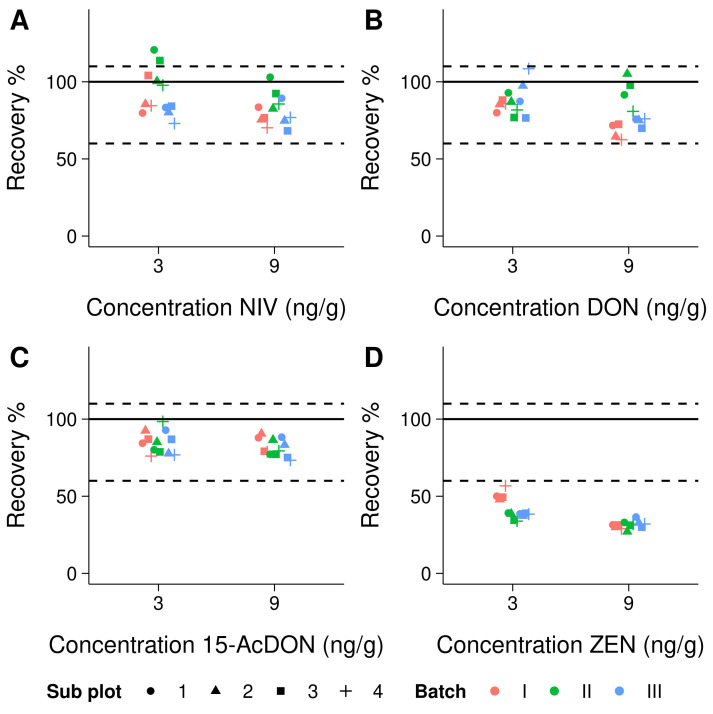
Recovery of nivalenol (NIV), deoxynivalenol (DON), 15-acetyl deoxynivalenol (15-AcDON), and zearalenol (ZEN) from a maize field soil provided by JKI Braunschweig, Germany. The dashed line refers to the required performance criteria according to Commission Regulation (EC) no. 401/2006 for DON 100–500 ng $g^{-1}$. Point shapes refer to the different sub plots where the samples were taken. Point colors refer to the respective extraction batch, processed on different days.

NIV, DON, and 15-AcDON met all validation criteria in the recovery experiment using JKI soil. A large variation of recovery was observed for almost all compound and concentration combinations, which was, on average, 3.14% larger compared to reference soils. Individual sub-plot soils were spiked to account for within-field variations and extracted in three batches on different days. A visual inspection and data analysis showed that NIV recovery was particularly clustered by the extraction batch (*p* < 0.001, F-ANOVA, df = 2) instead of the sub-plot (*p* = 0.146, F-ANOVA, df = 3), which indicates inter-day variability (Figure A2). However, there was no statistically significant effect on other mycotoxins.

Mean recoveries of NIV, DON, and 15-AcDON were not systematically related to soil organic carbon and clay content, respectively. Therefore, no significant effects of the respective contents on recovery were observed.

### 3.4. Limit of Detection and Quantification

The overall method LOD and LOQ obtained from the reference soils ranged from 0.11 to 0.33 ng $g^{-1}$ and 0.36 to 1.10 ng $g^{-1}$, respectively (Table A1). Highest mean LOQ was found for DON (0.8±0.1 ng g${}^{-1}$) followed by 15-AcDON (0.7±0.3 ng g${}^{-1}$) and NIV (0.7±0.2 ng g${}^{-1}$). The instrumental LOD and LOQ, measured in the mobile phase, ranged within 0.4–1.1 ng m$L^{-1}$ and 1.3–3.7 ng m$L^{-1}$, respectively. These parameters were clearly higher in matrix-matched and internal standard-corrected calibrations for recovery experiments with LOD and LOQ, ranging from 0.3 to 3.6 ng m$L^{-1}$ and 1.1 to 12.7 ng m$L^{-1}$, respectively. Translated into soil concentrations, matrix-matched calibration LOD and LOQ corresponds to 0.2–2.4 ng $g^{-1}$ and 0.7–8.4 ng $g^{-1}$, which is above the method LOD and LOQ. Instrumental limits of detection and quantification during method validation were estimated based on the parameters obtained from the calibration curve. However, this method was found to be too conservative, since signals can be visually identified at much lower concentrations. Therefore, instrumental LOD and LOQ in environmental sample assessments were calculated as the signal standard deviation of repetitive measurements of the smallest calibration standard (0.75 ng m$L^{-1}$, n > 7) multiplied with 3 and 10, respectively. This resulted in LOD and LOQ below the smallest calibration standard, which was further defined as the instrumental LOQ (0.75 ng m$L^{-1}$, 0.5 ng $g^{-1}$ soil).

### 3.5. Maize Field Soil Samples

**Figure 3:**
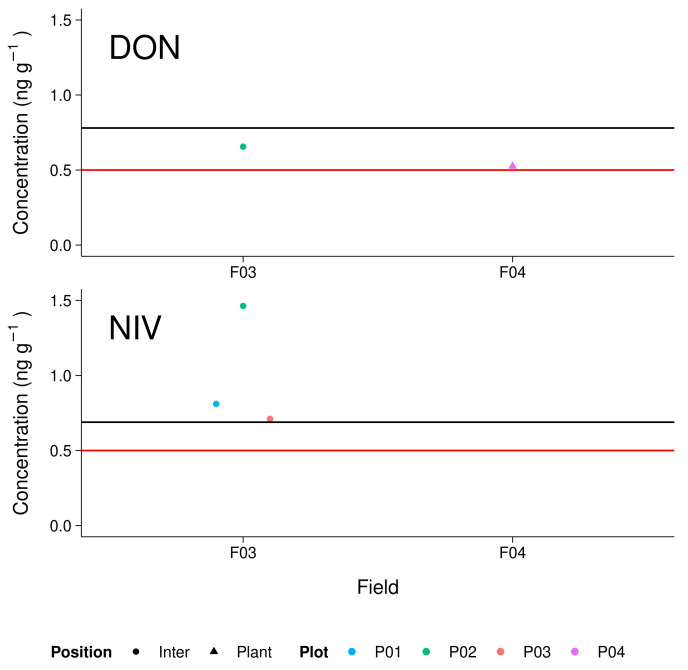
Levels of deoxynivalenol (DON) and nivalenol (NIV) found in pooled maize field soils. Red lines refer to the instrumental limit of detection, black lines refer to the mean extraction method limit of detection.

We report all data that were above the instrumental LOQ, i.e., 0.5 ng $g^{-1}$ soil, and mention the number of samples that were above the mean method LOQ (DON: 0.8 ng $g^{-1}$ soil; NIV: 0.7 ng $g^{-1}$ soil; Figure 3). Samples containing concentrations above the instrumental LOQ are further referred to as positive samples. We found 7.8% (5 out of 64) positive samples in the initial pooled sample testing. The positive samples originated from 2 of the 8 investigated fields (Field 3 and Field 4), with 4 positive samples from Field 3. Four of the five positive samples were collected between plant rows (“Inter”). However, due to the small number of positive samples, statistical evaluation of position effect was not possible.

DON was detected in the samples “Field 3 Plot 2 Inter” (0.7 ng $g^{-1}$) and “Field 4 Plot 4 Plant” (0.5 ng $g^{-1}$) at levels above the instrumental LOQ but below mean method LOQ. NIV exceeded instrumental and mean method LOQ in the samples “Field 3 Plot 1 Inter” (0.8 ng $g^{-1}$), “Field 3 Plot 2 Inter” (1.5 ng $g^{-1}$), and “Field 3 Plot 3 Inter” (0.7 ng $g^{-1}$). No sample contained 15-AcDON at levels above instrumental LOQ.

**Figure 4:**
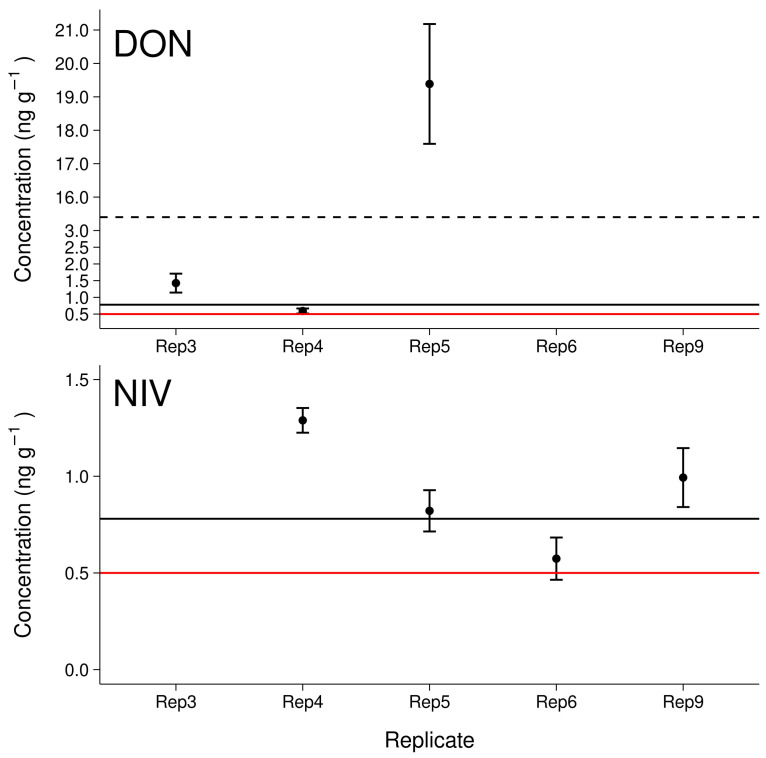
Levels of deoxynivalenol (DON) and nivalenol (NIV) found in single replicates of maize field soil samples. The red lines refer to the instrumental limit of detection, the black lines refer to the mean extraction method limit of detection. The dashed line highlights a break in the *y*-axis.

Since the pooled sample “Field 3 Plot 2 Inter” had the highest contents of both DON and NIV, it was selected for measurement of the individual samples to detect potential within plot concentration patterns, i.e., “hot-spots” or continuous levels (Figure 4). Three samples showed concentrations of DON that were above the instrumental LOQ, of which, two were above the mean method LOQ (Sample 3, 1.4±0.3 ng g${}^{-1}$; Sample 4, 0.59±0.08 ng g${}^{-1}$; Sample 5, 19±2 ng g${}^{-1}$). Overall mean concentration of DON in “Field 3 Plot 2 Inter” based on individual measurements was 2.3 ng $g^{-1}$, which is almost four-fold higher compared to the expected mean concentration based on the previously measured pooled sample. Concentrations of NIV were above the mean method LOQ in three of four positive samples: Sample 4, 1.29±0.06 ng g${}^{-1}$; Sample 5, 0.8±0.1 ng g${}^{-1}$; Sample 6, 0.6±0.1 ng g${}^{-1}$; Sample 9, 1.0±0.2 ng g${}^{-1}$. The overall mean concentration of NIV was 0.4 ng $g^{-1}$, which is one third of the expected concentration.

## 4. Discussion

### 4.1. Linearity and Matrix Effect

Linearity of the relevant working range was acceptable for the whole method validation process. No signal decrease for increasing concentrations was observed during visual inspection of the data, but WLS calibration was necessary in some cases where the measurement variation increased with concentration. In this study, linearity was tested in a comparably small range of 0.8–80 ng m$L^{-1}$ but based on available literature [17,18,19], soil mycotoxin concentrations can be expected to be mostly in the lower $ng\;g^{-1}$ range, and higher calibration concentrations are not of particular interest. Additionally, wider concentration ranges have already been tested elsewhere with good linearity [41].

Similar to our results, strong matrix effects have already been observed for other matrix-rich environmental samples, such as wastewater treatment plant effluent or agricultural drainage water, even when samples were cleaned via solid phase extraction [20,25]. In line with our findings, Schenzel et al. [25] observed a predominantly positive matrix effect for Type B trichothecenes, around 20–60%, but negative matrix effects were also shown for DON in aqueous environmental samples (−31 to 20%, [30]; −18 to −14%, [20]), and ZEN in soil (−8%) and sewage sludge (−49%, [16]). In a recent, very similar study, Kappenberg and Juraschek [55] showed only signal suppression ranging from −17 to −10% and −18 to −11% for DON and ZEN in various soils, respectively. The high variation in matrix effects observed in this study could not be attributed to the investigated physicochemical parameters of the soils. Similarly, the differences in Kappenberg and Juraschek [55] do not appear to be related to soil organic carbon content or soil texture. This is contrary to the expectations, since clay content and soil organic carbon represent the most important fraction for sorption sites and, thereby, the potential matrix load. Additionally, soil organic carbon content has been shown to be significant for matrix effects on steroid hormones and penicillin G, measured in LC–ESI–MS/MS [56]. However, soil organic carbon and clay content may be “too general” sum parameters to describe physicochemical properties of soil and a more detailed analysis of co-extracted molecules, such as organic acids and inorganic ions may provide better explanatory variables for matrix effects.

The high variation in matrix effects between soils and compounds observed here shows the importance of isotopically labeled internal standards in the analysis of mycotoxins in diverse soil matrices. Even the structurally very similar NIV and DON deviated in matrix effects by 15±7%. Correction with internal standards compensated the matrix effect for DON an NIV to less than 10% and, thus, to an acceptable range [57]. The usefulness of isotopically labeled internal standards has also been pointed out in other studies [20,30,31]. Additionally, the compound specific matrix effects show that there is a need for several analyte-specific internal standards. The remaining variation of our results after internal standard corrections can be explained with inter-day variability of laboratory work, which was also observed for batch-wise JKI soil extraction.

Assessing the matrix effect is of particular interest for analysis of soil samples since soils can have very different physicochemical properties, even on the field scale [27,28] and, therefore, matrices may also be different. Application of matrix-matched calibrations, as suggested by Zachariasova et al. [58], would be necessary for almost every sample where differences in matrices cannot be excluded. This would lead to an inflating number of measurements and costs. Our results show that it is possible to quantify different soil samples with the same external calibration curve, as long as proper isotopically labeled internal standards are included and all measurements belong to the same measurement batch.

### 4.2. Trueness and Precision

Recovery and repeatability were acceptable for NIV, DON, and 15-AcDON, with two exceptions at the lowest spiking level of LUFA2.4 for NIV and DON, respectively. In general, within and between soil variability appeared to increase with a decreasing spiking level while recovery decreased with spiking level. One reason could be the mismatch in internal standard concentration (25 ng m$L^{-1}$) and the lowest spiking level concentration (4.5 ng m$L^{-1}$) in the measurement. The ratio of signals may not be stable, leading to an overestimation of small concentrations. Furthermore, the soils were spiked level-by-level from small to high concentrations, and a systematic error cannot be excluded. Analysis of JKI soil recovery indicates that within-field variability may be less important than inter-day variability. Therefore, the recovery differences between reference soils may be rather attributed to the batch-wise extraction of respective reference soil samples than physicochemical soil differences. With an overall RSD_r_ of 10% for NIV, DON, and 15-AcDON, the variation was still in the required range [48].

The recovery and RSD_r_ of DON observed at the intermediate spiking level are very similar to the results that were recently published by Kappenberg and Juraschek [55] (concentration: 10 ng $g^{-1}$, recovery: 97–100%, RSD_r_: 6–9%) applying a similar extraction approach (79:20:1 acetonitrile/water/glacial acetic acid, ultrasonic bath for 1 h). However, the differences between the methods had a great effect on ZEN recovery, which was successfully extracted from the soil with satisfactory recovery and RSD_r_ by Kappenberg and Juraschek [55]. One reason may be the addition of glacial acetic acid, which has been reported to potentially improve the extraction process by breaking the interactions between mycotoxins and sample constituents (i.e., sugars and proteins) [59]. Furthermore, ZEN is stronger adsorbed to soil organic carbon [40], and a prolonged ultrasonic bath may be necessary for acceptable recovery rates.

While ZEN does not seem to be extractable by this method without modification, the method proposed in this study successfully validated two additional mycotoxins NIV and 15-AcDON. Furthermore, a recent study by Albert et al. [29], with a very similar acetonitrile:water approach, proved that this method is also suitable for analysis of aflatoxins in soil. This may be of particular interest, since climate change may cause a spread of *Aspergillus* to European regions where *Fusarium* is currently predominant [60,61].

Our results show that the applied method is able to extract trace levels of NIV, DON, and 15-AcDON from soil with satisfactory recovery and repeatability. We recommend choosing the internal standard concentration close to the mean of the expected concentrations. Extraction and measurement should be randomly distributed between batches to account for inter-day variability.

### 4.3. Limit of Detection and Quantification

**Figure A3:**
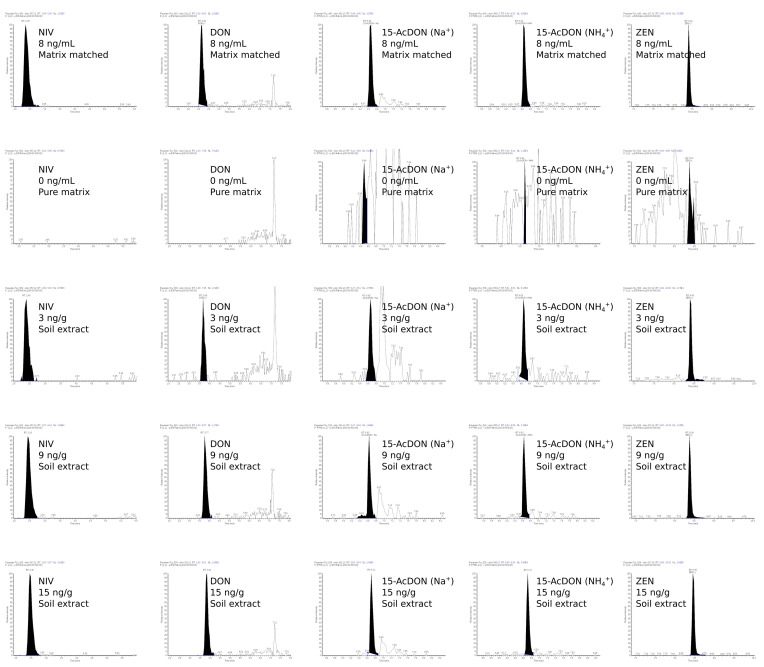
Example chromatograms of nivalenol (NIV), deoxynivalenol (DON), 15-acetyl deoxynivalenol (15-AcDON), and zearalenol (ZEN) measured in the matrix-matched calibration, pure matrix, and in soil extracts from RefeSol02A spiked at the levels 3, 9 and 15 ng $g^{-1}$ soil.

**Figure A4:**
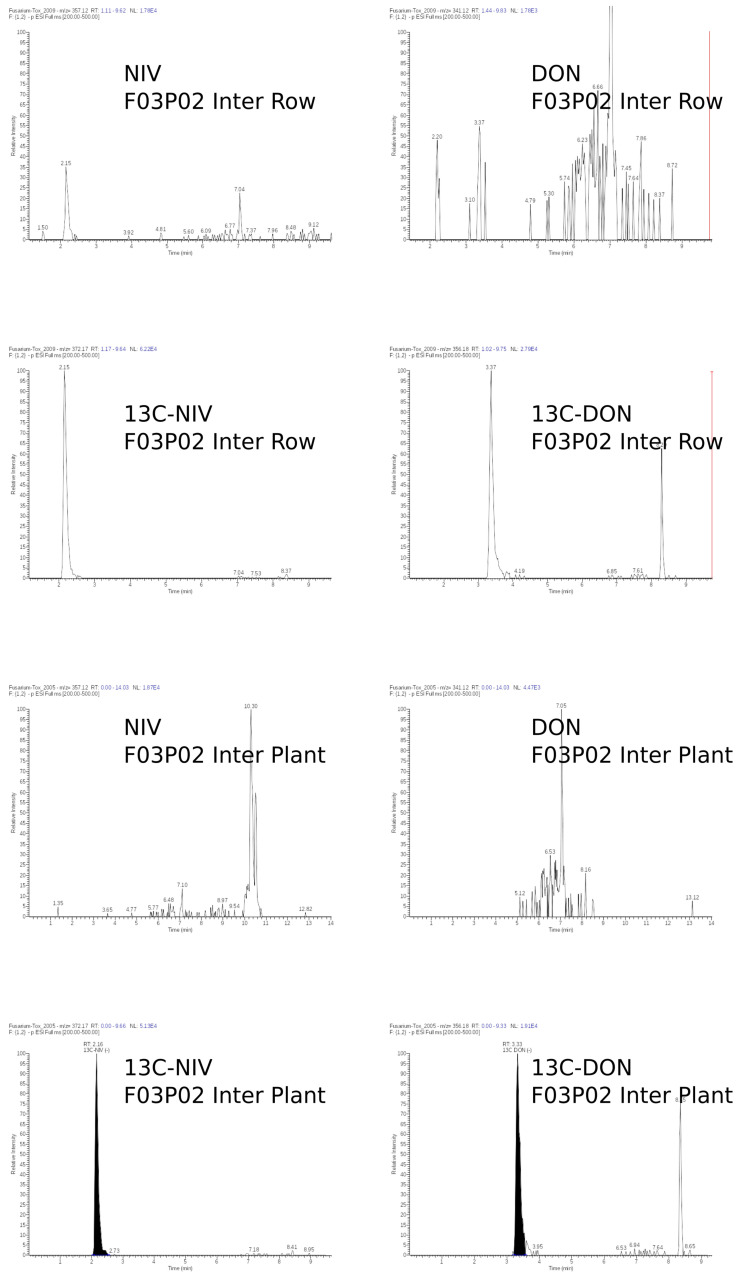
Example chromatograms of nivalenol (NIV, retention time 2.15 min), deoxynivalenol (DON, retention time 3.37 min), and respective stable isotope standards (13C-NIV, 13C-DON) measured in the maize field soil. Samples were taken from the same field and sub plot, but at different positions, i.e., between plants (inter plant) and between rows (inter row).

Estimates of the method LOD and LOQ were mostly below 1 ng $g^{-1}$, which indicates that the method potentially allows detection of NIV, DON, and 15-AcDON below the lowest spiking level of 3 ng $g^{-1}$. This is even below the instrumental limits estimated from the matrix-matched calibration curve, which were above 3 ng $g^{-1}$ in some cases and indicates that quantification was rather limited by calibration and instrumental performance than the extraction method. However, the measurements of the lowest levels were accepted here, since signals could be clearly identified and quantification matched with expected concentrations (Figure A3 and Figure A4). The comparatively high instrumental limits during method validation are attributed to the LOD and LOQ estimation method, which was based on the calibration curve. Therefore, the calibration was adapted for the second part of the environmental sample analysis. The calibration range was narrowed, the number of calibration levels increased, and the smallest calibration standard was measured repetitively. This allowed LOD and LOQ estimation based on “artificial” background noise close to the instrumental LOD, which were subsequently below the smallest calibration standard, which was further used as LOQ. The resulting instrumental and method LOD and LOQ are still in a similar range. To further develop this method, improved instrumentation with higher sensitivity is needed. One approach could be the usage of atmospheric pressure chemical ionization (APCI), which showed 12- and 10-fold higher signal, for NIV and DON, respectively [58], and 7.4-fold higher signals for DON and 15-AcDON in Jensen et al. [62].

### 4.4. Environmental Samples and Implications for Further Investigations

Our results show the presence of mycotoxins in 2 out of 8 field soils. Soil mycotoxin concentrations ranged from 0.53 to 19.38 ng $g^{-1}$ and 0.8 to 2.2 ng $g^{-1}$ for DON and NIV, respectively.

Occurrence of mycotoxins seemed to be more frequent between planting rows than individual plants. However, due to the low number of positive samples, there was no clear difference between the sampling positions, i.e., between plants and planting rows. If mycotoxins are washed off the plants during rain events, the rain water may distribute more homogeneously around the plants, leading to more widespread and homogeneous soil mycotoxin levels, but it is unclear whether mycotoxins are on the maize plant surface and accessible to rain. Although all above-ground maize plant parts can contain trichothecenes, the highest contents were found in rudimentary ears [39], which are physically protected from rain by husks. Therefore, washing off may not be the main route of mycotoxins from maize plants to the soil. Another path may be the translocation within the plant to the roots when above-ground parts are infected. Unfortunately, Schollenberger et al. [39] did not investigate natural maize root contents, but since trichothecenes were found in all parts of the plants, it seems likely that they are also present in plant roots. Furthermore, *Fusarium* can cause root rot, which can lead to trichothecene concentrations in the range 0.4–1.6 μg $g^{-1}$ [63] and 0.3–22.6 μg $g^{-1}$ [64] in root dry mass from wheat and maize, respectively. Consequently, the levels observed in this study may also originate from in situ biosynthesis. Further studies should identify the main introduction routes of mycotoxins from maize plants to soil and the potential spatiotemporal distribution from the rhizosphere to bulk soil. Fate and transport of mycotoxins within soil is scarcely investigated and the analytical method proposed in this study with a low LOQ may help to determine the mycotoxin pathways.

We observed a mismatch between concentrations in the pooled sample and mean of individually analyzed samples of DON and NIV, respectively. The mean DON concentration was higher than expected while NIV was lower. This indicates that soil mycotoxin levels may be heterogeneous on very small scales of 10 cm (two times the diameter of the sampling containers) and “hot-spot” concentrations may be restricted to areas smaller than 10 cm within soils. As mentioned before, infested roots may have a great contribution to soil trichothecene concentration. If the edge of an infested plant root zone was sampled, it is possible that the two samples at the same position had different contents of fine root material. Additionally, soil microbial communities may vary even on cm scales [28], which may explain why the pooled sample concentration is different from the mean of individual samples.

The apparent small scale heterogeneity has implications for soil sampling strategies. When aiming at a representative mean level on the field scale, which is necessary for field emission estimates, many individual samples collected from a fine mesh of sampling points are necessary. However, the pooling of many samples causes dilution of the concentrations. The method proposed in this study may help to overcome this issue, due to the low limits of quantification. Further method developments can improve the analysis, as for example using an APCI to achieve lower instrument limits [58]. Additionally, method limits can still be improved, for example by increasing the extract concentration in the evaporation step. A greater concentration step also increases matrix concentrations and may require further clean-up steps, such as immunoaffinity columns or solid phase extraction. The addition of acetic acid to the extraction solvent and a longer ultrasonic bath step allows to include ZEN in the analysis [55].

Based on our results, extrapolation to a mean field mycotoxin concentration is not possible or linked to a high level of uncertainty. In this study design, we aimed to identify “hot-spot” concentrations in the soil with a minimum number of measurements. The screening approach, with initial analysis of pooled sub-sets of samples, clearly reduced the number of potential hot-spot samples. Identifying the soil concentration range of trichothecenes is of ecological relevance, since the fate and effect of trichothecenes in the environment is still largely unknown and current studies report contrasting results. While no degradation of DON was observed 28 day after direct application to soil [55], there was a clear reduction within 21 day when applied as contaminated harvesting residues [22,65]. This indicatesthat the way in which mycotoxins are introduced to soil also affects their degradation. However, while degradation of pure DON in soil has not been proven yet, DON-degrading microbial cultures have been extracted from soil in several studies [66,67,68,69,70], which indicates that some soils may have a mitigating effect on soil mycotoxin pollution. These cultures were able to degrade DON within 48 h to 2 weeks with 3-epi-DON, de-epoxy-DON and 3-keto-DON being the main degradation products. These substances, as well as masked mycotoxins and temporal changes, were not assessed in this study. Therefore, the number of fields containing mycotoxins and related substance soils may be even higher than 25% found here (2 out of 8 fields). Further investigations should assess degradation rates and main degradation products in soil to estimate the potential of soil to mitigate environmental mycotoxin pollution.

## 5. Conclusions

In this study, we successfully validated an extraction and measurement method for the simultaneous measurement of NIV, DON, and 15-AcDON in agriculturally managed soils. The method was applied to provide first insights into natural mycotoxin levels in maize field soils. Mycotoxins were detected at varying levels, down to trace amounts, which shows that reliable methods are imperative for analysis of environmental soil samples. Furthermore, we showed that mycotoxin levels in soil can be highly variable, even on small scales. We therefore recommend a fine mesh of sampling points for mean field concentration estimates or “hot-spot” detection. An initial pooled sample analysis combined with a low method limit of detection can clearly reduce the number of measurements.

## Figures and Tables

**Table 1:** Physicochemical soil properties of reference soils (LUFA2.4, LUFA6S, RefeSol01A, RefeSol02A) and fresh soil samples. Soil texture classes are abbreviated with T/t = clay/clayey, U/u = silt/silty, S/s = sand/sandy, L/l = loam/loamy, 2 = slight, 3 = moderate, e.g., slight silty sand = Su2.

Soil	Sand	Silt	Clay	Soil Texture	Carbon Content	Soil pH
	%	%	%		%	
LUFA2.4	32.1	41.6	26.3	Lt2	1.78	7.4
LUFA6S	23.8	35.3	40.9	Lt3	1.99	7.2
RefeSol01A	74	19.8	6.2	Sl2	0.89	5.3
RefeSol02A	5.7	78.3	16	Ut3	1.04	6.6
JKI soil	63.5	32.5	4	Su3	0.75	Na
Field 1	92.5	5	2.5	Ss	1.42	6.6
Field 2	63.5	32.5	4	Su3	0.68	5.9
Field 3	31	65	4	Us	0.89	7.1
Field 4	80	17.5	2.5	Su2	0.54	5.7
Field 5	19	57.5	23.5	Lu	1.41	7.4
Field 6	19	57.5	23.5	Lu	2.22	7.2
Field 7	22.5	22.5	55	Tl	2.4	7.5
Field 8	Na	17.5	82.5	Tt	1.2	6.8

**Table 2:** Overview of the retention time, molecular mass, and the exact masses of the most intensive ions used for quantification.

Analyte	Retention Time	Molecular Weight	Adduct	Target Mass
	(min)	(Da)		(*m/z*)
NIV	2.2	312.32	[M + COOH]${}^{-}$	357.1195
DON	3.4	296.16	[M + COOH]${}^{-}$	341.1242
15-AcDON	6.45	338.35	[M + Na]^+^	361.1257
			[M + NH_4_]^+^	356.1705
ZEN	8.4	318.36	[M − H]${}^{-}$	317.1389
^13^C_15_-NIV	2.2	327.32	[M + COOH]${}^{-}$	372.1701
^13^C_15_-DON	3.4	311.21	[M + COOH]${}^{-}$	356.1750

## Data Availability

The data presented in this study are available upon request from the corresponding author.

## Abbreviations

The following abbreviations are used in this manuscript:

JKI	Julius Kühn Institute
NIV	nivalenol
DON	deoxynivalenol
15-AcDON	15-acetyl-deoxynivalenol
ZEN	zearalenone
LC	liquid chromatography
HRMS	high-resolution mass spectrometry
RSD_r_	relative standard deviation
HPLC	high pressure liquid chromatography
PET	polyethylene
LOD	limit of detection
LOQ	limit of quantification
QQ plot	quantile–quantile plot
WLS	weighted least squares
F-ANOVA	analysis of variance based on F-test
LME	linear mixed effect
APCI	atmospheric pressure chemical ionization
EU	European Union
EC	European Commission

## Appendix A

**Table A1:** Mean recovery (standard deviation in parentheses), method limit of detection (LOD), and method limit of quantification (LOQ) of deoxynivalenol (DON), nivalenol (NIV), 15-acetyl-deoxynivalenol (15-AcDON), and zearalenone (ZEN) extracted from four standard reference soils spiked with 3–15 ng $g^{-1}$ soil dry weight. LOD and LOQ were calculated according to the Eurachem guideline, based on standard deviation of ten independent sample extract measurements with a nominal concentration of 3 ng $g^{-1}$. The LOD and LOQ of ZEN were not determined (n.d.).

Compound	Soil	Recovery			LOD	LOQ
		3 ng $g^{-1}$	9 ng $g^{-1}$	15 ng $g^{-1}$	$ng\;g^{-1}$	$ng\;g^{-1}$
NIV	LUFA2.4	112 (9)	106 (5)	102 (4)	0.15	0.5
	LUFA6S	93 (4)	92 (8)	89 (3)	0.25	0.82
	RefeSol01A	104 (9)	94 (7)	90 (7)	0.25	0.84
	RefeSol02A	86 (6)	93 (4)	87 (5)	0.26	0.86
DON	LUFA2.4	112 (9)	109 (5)	105 (5)	0.24	0.79
	LUFA6S	104 (8)	93 (8)	87 (3)	0.12	0.41
	RefeSol01A	100 (10)	100 (5)	99 (6)	0.33	1.1
	RefeSol02A	89 (6)	95 (5)	89 (4)	0.28	0.92
15-AcDON	LUFA2.4	107 (5)	100 (6)	100 (3)	0.26	0.88
	LUFA6S	95 (9)	94 (4)	88 (3)	0.11	0.36
	RefeSol01A	100 (10)	97 (7)	94 (3)	0.18	0.58
	RefeSol02A	84 (4)	80 (4)	84 (4)	0.18	0.62
ZEN	LUFA2.4	40 (4)	37 (6)	39 (4)	n.d.	n.d.
	LUFA6S	16 (1)	15 (2)	16 (1)	n.d.	n.d.
	RefeSol01A	32 (2)	31 (1)	34 (2)	n.d.	n.d.
	RefeSol02A	45 (3)	35 (3)	34 (2)	n.d.	n.d.
